# The discovery of plastid-to-nucleus retrograde signaling—a personal perspective

**DOI:** 10.1007/s00709-017-1104-1

**Published:** 2017-03-23

**Authors:** Thomas Börner

**Affiliations:** Institute of Biology, Molecular Genetics, Humboldt University Berlin, Rhoda Erdmann Haus, Philippstr 13, 10115 Berlin, Germany

**Keywords:** Chloroplast signal, Plastid signal, Retrograde signaling, Chloroplast development, Regulation of gene expression

## Abstract

DNA and machinery for gene expression have been discovered in chloroplasts during the 1960s. It was soon evident that the chloroplast genome is relatively small, that most genes for chloroplast-localized proteins reside in the nucleus and that chloroplast membranes, ribosomes, and protein complexes are composed of proteins encoded in both the chloroplast and the nuclear genome. This situation has made the existence of mechanisms highly probable that coordinate the gene expression in plastids and nucleus. In the 1970s, the first evidence for plastid signals controlling nuclear gene expression was provided by studies on plastid ribosome deficient mutants with reduced amounts and/or activities of nuclear-encoded chloroplast proteins including the small subunit of Rubisco, ferredoxin NADP+ reductase, and enzymes of the Calvin cycle. This review describes first models of plastid-to-nucleus signaling and their discovery. Today, many plastid signals are known. They do not only balance gene expression in chloroplasts and nucleus during developmental processes but are also generated in response to environmental changes sensed by the organelles.

## Introduction

It was firmly established already in the early 1970s that plastids of algae like *Euglena*, *Chlamydomonas*, and *Acetabularia* and of higher plants contain their own genomes, RNA polymerase activity to transcribe their genes, as well as ribosomes, tRNAs, and aminoacyl tRNA synthetases for protein synthesis (Gibor and Granick 1964; Kirk 1971; Tewari 1971; Gillham 1974). Chloroplasts were found to contain multiple copies of their genomes in several nucleoids, the number of which increases with plastid size (Gunning 1965; Herrmann and Kowallik 1970; Kung 1977). Good evidence existed for chloroplast genes to code for chloroplast ribosomal RNA, tRNAs, and the large subunit of Rubisco (ribulose-1,5-bisphosphate carboxylase/oxygenase) (Kirk 1971; Chan and Wildman 1972; Blair and Ellis 1973; Börner 1973; Bogorad 1975). A chlorophyll-binding protein of photosystem I, chloroplast ribosomal proteins, and components of cytochrome complexes were also proposed to be encoded in chloroplast DNA (Armstrong et al. 1971; Herrmann 1971; Mets and Bogorad 1971). It has soon been realized, however, that the size of chloroplast genomes deduced from electron microscopic pictures and renaturation kinetics is much too small to code for all components of the organellar genetic machinery, the photosynthetic apparatus, and the other plastid-localized metabolic pathways (Kolodner and Tewari 1975; Kung 1977). First studies on chromoplast DNA revealed identical size and biochemical properties with chloroplast DNA (Herrmann 1972; Falk et al. 1974). The existence, in higher plants, of several types of plastids like chloroplasts, amyloplasts, chromoplasts, gerontoplasts (a term coined by Peter Sitte in Sitte 1977), or proplastids raised the question as to the role of plastid and nuclear genomes in cell differentiation, in particular, in the formation of the different plastid types (Sitte 1977). Is the plastid type signaled to the nucleus to control plastid-type specific gene activities? Several lines of evidence had already earlier indicated that a large number of genes required for chloroplast biogenesis and function reside in the nuclear genome (Kirk and Tilney-Bassett 1967). Data collected during the early years of the molecular biology of plastids indicated that the proteins of the chloroplast envelope, of photosystems I and II, of the chloroplast ribosomes, and even the subunits of Rubisco are encoded by both chloroplast and nuclear genes (Kirk 1971; Börner 1973; Bogorad 1975). This observation strongly suggested the existence of one or more mechanisms, which coordinate the activity of genes in the genomes of chloroplasts and nucleus. Is the developmental and/or metabolic status of the plastid/chloroplast signaled to the nucleus?—a question that is also connected to the much discussed problem of chloroplast autonomy (Tewari 1971).

Today, we know that the developmental and metabolic states of plastids, in particular of chloroplasts, but also of mitochondria, are signaled to the nucleus via different signaling molecules and signal chains. The activity of nuclear genes is modified in response to these organellar signals to adapt gene expression and other metabolic functions in the organelle, but also processes outside the plastids, to the requirements of development and of changes in the environment (Chan et al. 2016; Kleine and Leister 2016; de Souza et al. 2017). The processes of organelle-to-nucleus signaling are often referred to as ‘retrograde signaling’ and the regulation of organellar gene expression by nucleus-encoded proteins as “anterograde signaling” although the latter is (with the exception of a few cases) in a strict sense no “signaling” and should be better termed “anterograde control” or “anterograde regulation.” Organellar signals control transcription and posttranscriptional processes in the nucleo-cytoplasmic compartment and vice versa nuclear-encoded proteins execute and control gene expression at all levels in the organelles.

## Coordination of the expression of chloroplast and nuclear genes—first hypotheses

First ideas about the control of the expression of nuclear and chloroplast genes coding for chloroplast proteins were developed specifically for the subunits of Rubisco (often called “fraction I protein” at that time; Kawashima and Wildman 1970). John Ellis from the University of Warwick, Coventry, contributed together with his coworkers essential insights into the functional role of chloroplast ribosomes by identifying products of protein synthesis in isolated chloroplasts and etioplasts and by applying specific inhibitors of cytoplasmic or chloroplast protein synthesis. He observed that 2-(4-methyl-2,6-dinitroanilino)-N-methylpropionamide, at concentrations that inhibit rather specifically protein synthesis on cytoplasmic ribosomes, rapidly inhibits the synthesis of both the large and small subunits of fraction I protein on chloroplast and cytoplasmic ribosomes, respectively, in greening detached pea shoots. Based on this finding, Ellis proposed a model “for the co-operation of nuclear and chloroplast genomes in the synthesis of fraction I protein by supposing that the small subunit is a positive factor required for the initiation of either transcription of the messenger RNA for the large subunit or for its translation. This model predicts that inhibition of synthesis of small subunit on cytoplasmic ribosomes will lead to a rapid inhibition of synthesis of large subunit on chloroplast ribosomes” (Ellis 1975). Givan and Criddle (1972) proposed also a not further elaborated regulatory mechanism as possible explanation for their observation that inhibition of protein synthesis in the cytoplasm affected not only the formation of small but also of large subunits of Rubisco in *Chlamydomonas*.

Somewhat later, Ellis (1977) substantially extended his model by formulating two principles for the control of chloroplast protein synthesis: “*The multisubunit completion principle*”... states that the function of organellar ribosomes is to synthesize some subunits of multisubunit proteins, the other subunits being products of cytoplasmic ribosomes... There is no reported case of a complete protein being made by organellar ribosomes, and the multisubunit completion principle states that this does not occur. “*The cytoplasmic control principle*”... states that cytoplasmic products control organellar protein synthesis, but that the converse does not occur... Combining these two principles leads to the suggestion that the cytoplasmic products, which control the synthesis of subunits in the organelle, are the same subunits which combine with them to form the complete protein. This is not a necessary conclusion, but it describes the most economical arrangement” (Ellis 1977).

A study on the incorporation of (^14^C) leucin into total plastid protein of *Euglena gracilis* cells growing at 27 or 35 °C without or with chloramphenicol or cycloheximide in the medium led to the postulate that proteins synthesized on plastid ribosomes have an influence on the translation in the cytoplasm and vice versa (Brandt 1976). This investigation had several weaknesses and has not been discussed by other authors in the following years.

## Expression of nuclear genes for plastid proteins is reduced in plastid ribosome deficient mutants—evidence for plastid-to-nucleus signaling

Studies on the *Pelargonium* cultivar “Mrs. Parker” provided a first hint of a role of plastids in the coordination of gene activities in the nucleus and chloroplasts, i.e., that in a certain sense the converse of the “cytoplasmic control principle” might happen. This cultivar is a chimera characterized by green leaves with white margins. It eventually develops entirely green and white leaves containing normal wild-type chloroplasts and undifferentiated mutant plastids, respectively. The cultivar “Mrs. Parker” was the subject of my doctoral studies in Rudolf Hagemann’s lab at Martin Luther University in Halle (Saale), East Germany. I could not detect plastid rRNAs in the white leaves of “Mrs. Parker” and somewhat later not in white leaves of several other *Pelargonium* cultivars, an observation that was supported by the lack of ribosomes in electron microscopic pictures of the mutant plastids. The plastid ribosome deficiency should lead to the inability of the mutant plastids to express their protein-encoding genes and made this mutant an ideal object of investigations into the functional role of plastid ribosomes (Börner et al. 1972, 1973). The protein with the best evidence for being synthesized on chloroplast ribosomes was the large subunit of Rubisco at that time (Criddle et al. 1970; Chan and Wildman 1972; Blair and Ellis 1973). To gain further support for the supposed lack of ribosomes in the *Pelargonium* mutant plastids, I isolated therefore soluble proteins from green and white leaves and separated them on native polyacrylamide gels. This method allows for the easy detection of ‘fraction I protein’ (Rubisco). As anticipated, a protein band at the position of fraction I protein consisting of the large and small Rubisco subunits was found in preparations of soluble proteins from green but not from white leaves. The missing Rubisco band was obviously due to the incapacity of ribosome-deficient plastids to synthesize the large subunits. However, since the small subunits of Rubisco are synthesized on cytoplasmic ribosomes, which are functioning in the white leaves, the obvious lack of a corresponding band in the gels was not expected (Börner et al. 1974).

The lack of the small Rubisco subunits in the mutant plastids of “Mrs. Parker” and of other cultivars could have different reasons. One would be that proteases degrade the small subunits because they are not protected by assembling with the large subunits to form an active enzyme complex. Another reason could be a specific mechanism that coordinates the synthesis of the two Rubisco subunits as suggested by Givan and Criddle (1972) for *Chlamydomonas* and later by Ellis (1975) for higher plants. I hypothesized, however, that there might exist a more general mechanism that coordinates the expression of chloroplast and nuclear genes, i.e., a mechanism that would, in white “Mrs. Parker” leaves, repress or not activate the expression of most or all nuclear genes coding for those proteins that are specifically needed in photosynthetically active chloroplasts.

To rule out the possibility that just degradation of the small subunits is the reason for their absence in plastid ribosome deficient mutant leaves, I decided to determine the activity of such chloroplast-localized enzymes which were known to be synthesized on cytoplasmic ribosomes and would, in contrast to the small Rubisco subunits, not form complexes with other proteins encoded in chloroplast genes and synthesized on chloroplast ribosomes. Unfortunately, *Pelargonium* leaves (green leaves more, white ones somewhat less) turned out to contain substances, most likely polyphenols, which precipitate and inactivate proteins during the isolation procedure. By modifying the conditions, I was able to keep proteins soluble but not to preserve their activities (Börner et al. 1974). Therefore, I studied albino mutants of other species in 1972 and 1973 and luckily found two barley (*Hordeum vulgare* L.) mutants, *Saskatoon* and *albostrians*, to lack plastid ribosomes and the large and small subunits of Rubisco (Hagemann et al. 1973; Börner et al. 1976). Using sensitive immmunoelectrophoresis, we detected traces of Rubisco in young white leaves of “Mrs. Parker” but not in light-grown or etiolated albostrians leaves suggesting residual translational activity in plastids of the *Pelargonium* mutant and the complete absence of ribosomes in plastids of white leaves of the barley mutant (Börner et al. 1976; Hagemann and Börner 1978; Reichenbächer et al. 1978). Activity tests of Rubisco supported also its absence from white leaves of albostrians and Saskatoon. For further studies, I selected the ferredoxin: NADP^+^ reductase and the Calvin cycle enzyme phosphoribulokinase. Like the small subunit of Rubisco, these enzymes were proposed to be synthesized on cytoplasmic ribosomes (Armstrong et al. 1971; Ellis and Hartley 1971; Vaisberg et al. 1976). In contrast to the small Rubisco subunit, however, ferredoxin: NADP reductase (at least its soluble form) and phosphoribulokinase were not known to form part of complexes with other proteins. A poorly reacting antibody against ferredoxin: NADP^+^ reductase from *Antirrhinum majus* did not detect ferredoxin: NADP^+^ reductase in white leaves. Activity tests revealed later clearly the existence of this enzyme in white leaves, though with reduced activity; white leaves contain about 60% of the soluble form and less than 10% of the membrane-bound form of the ferredoxin: NADP^+^ reductase activity compared with green leaves (part of these results were later published in Börner 1981). The activity of phosphoribulokinase was measured together with Klaus-Peter Rindt, who at the time was working on enzymes involved in bacterial photosynthesis in Erich Ohmann’s group at the Institute of General Botany of Martin Luther University. The results of this preliminary study suggested a marked reduction of this enzyme in white primary leaves of albostrians barley, a reduction being much more drastic than in the case of the ferredoxin reductase. Since all these observations supported the hypothesis of a controlling influence of the plastid/chloroplast on the expression of nuclear genes coding for chloroplast proteins, I was already in the mid 1970s convinced that such a regulatory mechanism really operates in leaves.

Quite surprisingly, I got the permission to travel to the UK in March and April 1977 to visit several labs involved in research on chloroplasts and plant mitochondria. This was rather exceptional for a young scientist in East Germany and only possible thanks to the strong support by my PhD supervisor and head of the Institute of Genetics at the University of Halle, Rudolf Hagemann (and certainly thanks to the fact that my wife and our two daughters would stay at home as a guarantee for my return to East Germany). Most of the time I spent in the lab of William Bradbeer at the King’s College, University of London. Bill was an expert of the biochemistry and physiology of chloroplast development including the Calvin cycle. I brought seeds of Saskatoon and albostrians with me to London, and Bill selected two Calvin cycle enzymes, phosphoribulokinase and glyceraldehyde 3-phosphate dehydrogenase (NADP^+^), to be studied in fractions of soluble proteins from green and white leaves of the barley mutants. In addition, the activities of the cytoplasmically localized isoenzyme, glyceraldehyde 3-phosphate dehydrogenase (NAD^+^), and of phosphoglycerate kinase were determined. The measured activity of phosphoglycerate kinase originated from two isoenzymes, one located in the plastids, the other in the cytoplasm. Already the first series of experiments clearly demonstrated extremely low activities of the plastid-localized enzymes phosphoribulokinase and glyceraldehyde 3-phosphate dehydrogenase (NADP^+^) in white leaves of albostrians while the cytoplasmic enzyme glyceraldehyde 3-phosphate dehydrogenase (NAD^+^) and the jointly measured activities of the plastid and cytoplasmic forms of phosphoglycerate kinase were much less affected. Similar results were obtained with Saskatoon and irrespective of whether etiolated or illuminated seedlings were investigated*.* These experiments were continued by Yvonne Atkinson in Bill’s lab.

## Plastid RNA as derepressor of nuclear genes?

Shortly, after my visit to the UK, I presented a poster during the International Conference on the Regulation of Developmental Processes in Plants, which was held in July 1977 in Halle. The poster reported results of studies performed in Halle on the large and small subunits of Rubisco, phosphoribulokinase, and ferredoxin: NADP^+^ reductase together with the hypothesis of regulatory effects of the plastid/chloroplast on the expression of nuclear-encoded plastid proteins (Börner 1977). Based on genetic analyses, the barley mutant alleles Saskatoon and albostrians were originally described as examples of so-called plastome mutator genes, i.e., nuclear alleles that cause mutations in the plastid DNA (Arnason and Walker 1949; Hagemann and Scholz 1962). In contrast to several other plastome mutator genes, which generate various plastome mutations with different phenotypes (reviewed in Börner and Sears 1986), the two barley alleles were thought to induce in each generation the same mutant phenotype characterized by white leaves with undifferentiated, ribosome-deficient plastids (Börner et al. 1976). It is difficult to imagine how a nuclear gene product might induce plastome mutations leading again and again to an identical phenotype. Yet the mutations could theoretically be very drastic alterations of the genome like large deletions similar to what is known from albino seedlings generated by microspore culture (Day and Ellis 1984; Harada et al. 1991), massive alterations in the DNA sequence, or the complete loss of chloroplast DNA. Yet earlier investigations had shown that DNA is present in ribosome-free albostrians plastids (Hagemann and Börner 1978) and later we could exclude large deletions, insertions or rearrangements (Hess et al. 1993). Alternatively, the albostrians and Saskatoon gene products might not induce plastome mutations but be proteins that are needed for the biogenesis and function of chloroplast ribosomes. Once lacking, ribosomes cannot reappear since they depend on their own protein synthesizing capacity to produce the proteins they are composed of, i.e., the lack of chloroplast ribosomes should be stably inherited like a genuine DNA mutation (Börner and Sears 1986; Zubko and Day 1998). We know today that the albostrians allele does indeed not act as plastome mutator, but prevents the formation of plastid ribosomes early in plant development (M. Li, N. Stein, T. Börner, unpubl. data) and the same is probably true for Saskatoon (see also Han et al. 1992 for a similar mutant in maize). In 1977, however, I considered both a mutation in plastid DNA and the lack of ribosomes as equally likely primary cause of the albostrians phenotype. Therefore, my poster presented a hypothetical model in which an altered plastid gene product or its absence affects the expression of nuclear genes coding for chloroplast proteins.

Curious though, Jürgen Feierabend, then at the Ruhr University Bochum, showed during the same conference contrasting results on a poster directly adjacent to my presentation (Feierabend 1977a). Feierabend and coworkers published shortly before the conference that growth at elevated temperature (32 °C) prevents the formation of plastid ribosomes in leaves of rye (*Secale cereale* L.) (Feierabend and Schrader-Reichhardt 1976; Feierabend and Mikus 1977). The plastid ribosome deficient leaves are chlorotic; hence, these plants represent phenocopies of the barley mutant albostrians and Saskatoon. Yet, in spite of the chloroplast ribosome deficiency, the chlorotic rye leaves do not exhibit the strikingly low amounts and activities of nuclear-encoded plastid proteins that we have observed in the barley mutants. Chlorotic rye leaves grown at elevated temperature and under illumination contain the small subunit of Rubisco in the absence of the large subunit. Related to the activities in green leaves grown at permissive temperature (22 °C), the plastid ribosome deficient leaves exhibit nearly normal activities of 97% (glyceraldehyde 3-phosphate dehydrogenase (NADP+)) and 83% (phosphoribulokinase). In addition, other plastid enzymes are present with virtually normal activities (Feierabend 1977a; Feierabend and Schrader-Reichhardt 1976).

In the light of the results reported by Feierabend und coworkers on plastid ribosome deficient rye, it seemed unlikely that the ribosome deficiency and impaired protein synthesis in the mutant barley plastids (or any component/process that dependents upon plastid protein synthesis) would be responsible for the extremely low amounts and activities of nuclear-encoded plastid proteins in albostrians and Saskatoon. We speculated, therefore, that mutation of the plastid DNA in albostrians and Sakatoon barley might affect nuclear gene expression independently of plastid protein synthesis via altered plastid transcript(s). According to our hypothesis, one or more “derepressors” would be transcribed from plastid DNA (implying that the “derepressors” are plastid RNAs) starting with the beginning of chloroplast development. The “derepressor” would derepress/activate those nuclear genes which code for proteins that are specifically needed in photosynthetically active plastids and would be present in wild-type leaves, also when grown at elevated temperature, but not in white leaves of albostrians barley. Bill Bradbeer presented this hypothesis together with the results of our investigations into enzyme activities in green and white leaves of albostrians and Saskatoon during the International Symposium on Chloroplast Development organized by George Akoyunoglou and Joan Argyroudi-Akoyunoglou and held in July 1978 on the island of Spetsai (Bradbeer and Börner 1978). The idea of RNA transcribed from plastid genes but functioning outside the plastids got support in the same year by a publication in *Nature* reporting that several tRNA species have their genes within the chloroplast DNA of *E. gracilis* but are involved in protein synthesis on cytoplasmic ribosomes (McCrea and Hershberger 1978). We included therefore the possibility that plastid-derived RNA controls protein synthesis on cytoplasmic ribosomes in the publication of our ideas about regulatory plastid RNA involved in the transcription of nuclear genes (Bradbeer et al. 1979). Not necessarily the existence of a regulatory plastid RNA, but the idea of an involvement of plastid transcription in signaling to the nucleus got later support by experiments with tagetitoxin and rifampicin, inhibitors of the plastid-encoded RNA polymerase (Lukens et al. 1987; Rapp and Mullet 1991; Woodson et al. 2013), and with nalidixic acid, a prokaryotic DNA gyrase inhibitor that affects plastid DNA replication and transcription (Gray et al. 1995), as well as by the analysis of Arabidopsis mutants defective in plastid sigma factors SIG2 and SIG6 (Woodson et al. 2013).

The contrasting results obtained with ribosome deficient plastids of heat-treated rye seedlings and of barley mutants, respectively, triggered, together with the “regulatory-RNA-hypothesis”, investigations into transcription of plastid DNA in this material. Although to different extent, either type of ribosome-deficient plastids synthesizes RNA (Bünger and Feierabend 1980; Siemenroth et al. 1981), i.e., a general lack of transcription of plastid genes in one type was not the reason for the observed differences. However, these studies revealed the existence of a nuclear-encoded plastid RNA polymerase (later dubbed *NEP* by Hajdukiewicz et al. 1997) and plastid rRNA was the first identified product of NEP (Siemenroth et al. 1981).

I moved to the Humboldt University at Berlin in 1982 and established there a lab dealing with research on the molecular genetics mainly of plants and cyanobacteria. This was a difficult and tedious task under the East German conditions. Therefore, we could resume research on albostrians barley only at the end of the 1980s and then more extensively after the reunification of the two Germanys. The problem of plastid-to-nucleus signaling became the topic of Wolfgang Hess’ PhD and Habilitation theses.

Intermediates of chlorophyll biosynthesis were among the factors first suggested to be involved in plastid-to-nucleus signaling (Johanningmeier and Howell 1984; Kropat et al. 1997). The biosynthesis of tetrapyrroles including chlorophyll, heme, siroheme, and phytochromobilin starts in plants with the ligation of glutamate to tRNA^Glu^ (Brzezowski et al. 2015). Since tRNA^Glu^ was known to be encoded by a chloroplast gene (Hollingsworth and Hallick 1982; Kuntz et al. 1984), we regarded it as a candidate for a plastid RNA involved in plastid-to-nucleus signaling. This RNA would not need to leave the chloroplast as originally thought (Bradbeer and Börner 1978; Bradbeer et al. 1979) but act inside the plastids via its role in tetrapyrrole synthesis. In collaboration with Gaby Walter and Paul Hoffmann (and later Bernhard Grimm) from our University and with Wolfhart Rüdiger from the Ludwig Maximilian University at Munich, we studied the accumulation of tRNA^Glu^ and the activity of several enzymes of the tetrapyrrole pathway in leaves of albostrians barley and in leaves of rye grown at elevated temperature. A remarkable difference between the two types of plastid ribosome deficient material was the extremely low accumulation (not detected by RNA blot hybridization, only by PCR) of tRNA^Glu^ in white albostrians leaves while the chlorotic rye leaves contained substantial amounts of this RNA (Hess et al. 1992a, b; Walter et al. 1995). This difference is certainly caused by the lack of ribosomes in albostrians plastids, as opposed to the presence of a few ribosomes in the plastids of heat-treated rye leaves (Feierabend and Schrader-Reichhardt 1976; Feierabend 1977b). Due to the lack of ribosomes, the plastid-encoded plastid RNA polymerase (PEP) cannot be synthesized and only NEP transcribes the plastid genes in white albostrians leaves (Hess et al. 1993; Zhelyazkova et al. 2012). In contrast, NEP should be active together with the plastid-encoded plastid RNA polymerase (PEP) in the chlorotic rye leaves as suggested by the similar amounts of uridine incorporation into RNA of the plastids at 32 °C and chloroplasts at 22 °C (Bünger and Feierabend 1980). The plastid *trnE* gene coding for tRNA^Glu^ is transcribed mainly by PEP and only very weakly by NEP (Hanaoka et al. 2003; Zhelyazkova et al. 2012). Thus, the lacking PEP activity will result in the observed low levels of tRNA^Glu^ in white albostrians leaves, which in turn might be one reason for the much lower content of chlorophyll and carotenoids in albostrians plastids vs. plastids of heat-treated leaves (Börner and Meister 1980; Yaronskaya et al. 2003; Feierabend 1977b). Even though protein synthesis does not compete with tetrapyrrole biosynthesis for tRNA^Glu^ in the ribosome-free albostrians plastids, the very low quantity of tRNA^Glu^ will limit the biosynthesis of tetrapyrroles (Yaronskaya et al. 2003). The common precursors of all tetrapyrroles are channeled in the direction of heme synthesis in albostrians plastids while the formation of chlorophylls is repressed. In addition to other effects, we observed reduced amounts of Mg-chelatase contrasted by a drastically enhanced accumulation of Fe-chelatase (Yaronskaya et al. 2003).

There is now experimental evidence for a positive effect of increased flux through the heme branch of the tetrapyrrole pathway on the transcription of photosynthesis-associated nuclear genes (PhANGs) suggesting that heme might leave the chloroplast and act as a signal molecule (Woodson et al. 2011). Heme might not act as a positive signal in ribosome-free albostrians plastids, since the content of non-covalently bound heme is diminished to only 55 and 38% in etiolated and light/dark-grown white compared with green leaves in spite of the enhanced levels of Fe-chelatase. We concluded that altered levels of intermediates and products of the tetrapyrrole pathway—which results at least in part from the impaired transcription of the *trnE* gene—might be involved in plastid-to-nucleus signaling (Yaronskaya et al. 2003). Recent studies on sigma factor mutants in Arabidopsis confirm a role of *trnE* transcription in plastid-to-nucleus signaling and PhANG expression (Woodson et al. 2013). Sigma factor SIG2 supports transcription of *trnE* by PEP (Hanaoka et al. 2003). Woodson et al. (2013) observed that in an Arabidopsis *sig2* mutant the levels of tRNA^Glu^, non-covalently bound heme, and PhANG transcripts were low—a situation similar to what we found in white leaves of albostrians. PhANG expression was restored to control levels by heme overexpression and in a *gun1sig2* double mutant (Woodson et al. 2013). GUN1 is a chloroplast-localized PPR protein. Lack of functional GUN1 restores PhANG transcription in albinotic leaves treated with norflurazon or lincomycin (Nott et al. 2006).

## Plastid proteins, metabolites, and hormones as “plastid factors”

The idea of a “derepressor” RNA transcribed from plastid DNA, leaving the plastids and moving to the nucleus to activate silenced genes (Bradbeer and Börner 1978; Bradbeer et al. 1979) originated from the contradictory results obtained with plastid ribosome deficient rye seedlings grown at elevated temperature and plastid ribosome deficient seedlings of mutant barley lines as outlined above. It was obvious that there could be reasons other than mutant chloroplast DNA in albostrians vs. wild-type chloroplast DNA in rye for the low activities of nuclear-encoded plastid enzymes in barley vs. nearly normal activities in heat-treated rye seedlings: species-specific effects, the presence of ribosomes in heat-treated chlorotic rye leaves, or the developmental stage at which the impaired plastid translation becomes effective. A species-specific difference in the response to plastid ribosome deficiency between barley and rye has been ruled out by studies on barley seedlings grown at elevated temperature (Allsop et al. 1981). Another potentially important difference between the mutants and plants grown at elevated temperature is that the white leaves of albostrians (Saskatoon has not been investigated to comparable extent) do not contain Rubisco (Reichenbächer et al. 1978) while the chlorotic rye leaves show residual Rubisco activity (8% of the activity measured in extracts of green leaves of seedlings grown at permissive temperature and under light; Feierabend and Schrader-Reichhardt 1976), i.e., the chlorotic leaves must possess a few plastid ribosomes as noted by Feierabend and Schrader-Reichhardt (Feierabend and Schrader-Reichhardt 1976; Feierabend 1977b). It has been shown much later that an impaired but not fully blocked translation in plastids due to mutation of the nuclear gene for a plastid ribosomal protein (PRPL11) has no effect on the expression of PhANGs (Pesaresi et al. 2006). Moreover, the lack of ribosomes in albostrians barley is induced already at early stages of embryo development whereas the ribosome deficiency in rye is initiated only during germination at elevated temperature and fully effective later in leaf development (Hagemann and Scholz 1962; Feierabend and Schrader-Reichhardt 1976). An effect of plastid translation on nuclear gene expression has been observed also by use of inhibitors, such as lincomycin, streptomycin, and chloramphenicol. Inhibition of translation in plastids affected nuclear gene expression only if applied within the first 2–3 days of seedling development in mustard or tobacco (Oelmüller et al. 1986; Bajracharya et al. 1987; Gray et al. 1995), i.e., at a time point when the plastids in heat-treated rye leaves should still have a substantial quantity of active ribosomes. Thus, the existence of plastid ribosomes and/or the function of plastid protein synthesis early in leaf development might be responsible for the observed missing or small effects on nuclear gene expression in heat-treated rye seedlings. Consequently, one has to consider also factors other than RNA acting as “plastid factors” or “plastid signals” in plastid-to-nucleus signaling.

The evolutionary origin of the chloroplast genome was another reason to entertain doubts about the existence of a chloroplast gene encoding an RNA (or protein) involved in the regulation of gene expression in the nucleus. The hypothesis about the evolution of chloroplasts from cyanobacteria (Mereschkowski 1905; Margulis 1970) triggered an intense debate and the generation of alternative hypotheses in the 1970s (e.g., Raff and Mahler 1972; Bogorad 1975). Rudolf Hagemann organized in 1977 an international meeting to discuss the various conceptions about the evolution of eukaryotic cells. It was Peter Sitte (Sitte 1981) who convinced me of the endosymbiotic origin of chloroplasts and mitochondria by his talk during this meeting where he discussed phylogenetic relationships based on sequences of proteins and nucleic acids from chloroplasts, nuclei, and bacteria in a similar way as was published somewhat later by Schwartz and Dayhoff (1978) and Doolittle and Bonen (1981). If the chloroplast genes have evolved from cyanobacterial genes, then it was not easily understandable how these organelles could possess a gene for a regulator of nuclear gene activities. There is certainly the possibility that a chloroplast gene got another or an additional function during evolution. Nevertheless, the endosymbiosis theory weakened our idea from a chloroplast “derepressor” RNA.

I did not get the permission from the responsible East German authorities to take part together with Bill Bradbeer in the 1978 Spetsai meeting on Chloroplast Development, but Rudolf Hagemann did. Hagemann’s talk during this meeting was devoted to our studies on plastid ribosome deficient mutants of *Pelargonium* and barley. Because of the reasons outlined above, we decided, in agreement with Bill Bradbeer, that Hagemann should also shortly comment in his talk on the low activities and levels of nuclear-encoded chloroplast proteins in the barley mutants and point out that “it has still to be analyzed whether the postulated ‘chloroplast control principle’, i.e., a control by plastids of nuclear gene expression complementing the ‘cytoplasmic control principle’ formulated by Ellis (1977), is exerted by RNA or ‘derepressor’ proteins or mechanisms not necessarily based on RNA or proteins, e.g., by plant hormones” (Hagemann and Börner 1978).

**Fig. 1:**
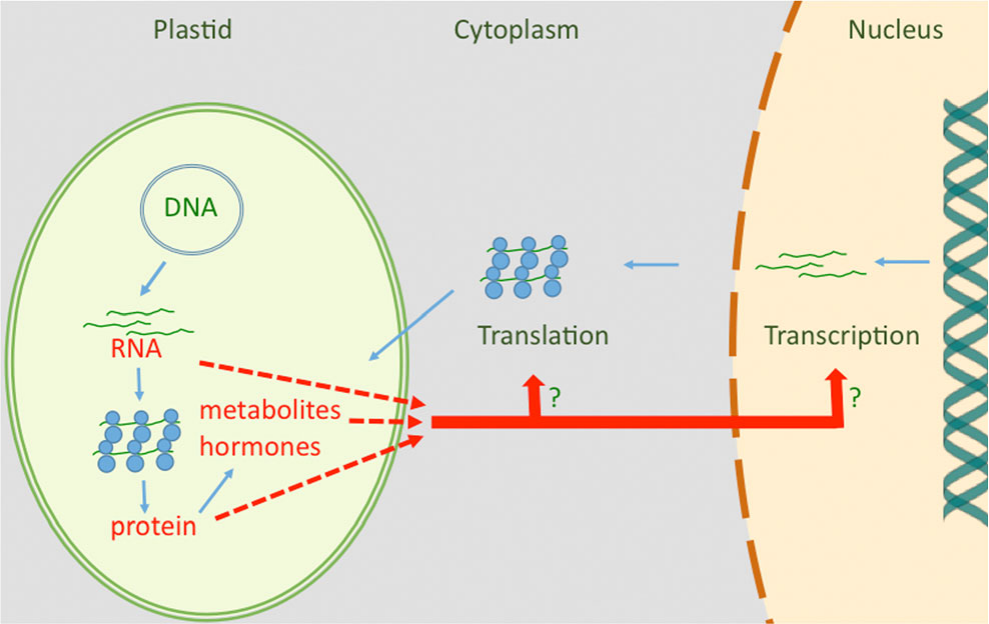
RNA, protein, metabolites, or hormones might act as plastid signals and control the expression of nuclear genes at the level of transcription or translation. This model reflects the hypotheses developed on the basis of reduced amounts and activities of nuclear-encoded chloroplast proteins in barley mutants (Bradbeer and Börner 1978; Hagemann and Börner 1978; Bradbeer et al. 1979). That plastid signals affect nuclear gene expression already at the level of transcription was shown by studies on *LHCP* transcription and transcript levels in albinotic mutants and norflurazon-treated seedlings in the mid of the 1980s (Mayfield and Taylor 1984; Batschauer et al. 1986; Oelmüller and Mohr 1986)

In 1978, we regarded plastid RNA, proteins, hormones, and other metabolites as alternative candidates for a plastid factor controlling PhANG expression in the nucleo-cytoplasmic compartment (Bradbeer and Börner 1978; Hagemann and Börner 1978; Fig. 1). RNA remained our favorite (Bradbeer et al. 1979), but only for a short time. Research over the last 30 years on plastid-to-nucleus signaling has made it likely that all these types of molecules originating from plastids/chloroplasts might play roles in plastid-to-nucleus signaling (Chan et al. 2016; Kleine and Leister 2016; de Souza et al. 2017).

## Support for the hypothesis of plastid-to-nucleus signaling from further studies during the 1980s

A couple of years later, the idea of signals from plastids to nucleus got support from studies on albino mutants and plants treated with herbicides or inhibitors of chloroplast transcription and translation, which demonstrated that impaired chloroplast development reduces the steady state levels of mRNAs transcribed from PhANGs (most analyzed were *LHCP* transcripts) and the transcription of PhANGs as demonstrated by run-on assays (Harpster et al. 1984; Mayfield and Taylor 1984, 1987; Batschauer et al. 1986; Oelmüller and Mohr 1986; Oelmüller et al. 1986; Burgess and Taylor 1988; Giuliano and Scolnik 1988).

Similar to growth under elevated temperatures, treatment of seedlings with inhibitors of carotenoid biosynthesis, like the herbicide norflurazon, results in chlorosis and chloroplast ribosome deficiency (Bartels et al. 1967). In contrast to heat-treated seedlings, but like albostrians and Saskatoon, the herbicide-treated seedlings of rye and mustard (*Sinapis alba* L.) are characterized by extremely low glyceraldehyde-phosphate dehydrogenase (NADP^+^) activities (Feierabend and Schubert 1978; Reiss et al. 1983). Moreover, the activities of the peroxisomal enzymes glycolate oxidase and hydroxypyruvate reductase are very low. Since the effects on plastid and peroxisomal enzymes could only be observed in seedlings grown under white light but not in dark-grown seedlings, they were regarded as consequences of photodamage of the proteins due to the absence of protective carotenoids and not discussed under the aspect of plastid signals to the nucleus (Feierabend and Kemmerich 1983; Reiss et al. 1983). Feierabend and Kemmerich (1983) hypothesized that lipid peroxidation specifically transmits photooxidative damage via membrane contacts from plastids to peroxisomes. Later, it was shown that the expression of nuclear genes for the peroxisomal glycolate oxidase, catalase and hydroxypyruvate reductase is indeed under control of plastid signal(s) (Schwartz et al. 1992; Boldt et al. 1997).

Reviewing research on regulatory interactions between nuclear and plastid genomes from the first reports on albostrians barley until the end of the 1980s, William Taylor (1989) speculated “What sort of molecule might the chloroplast signal be?...It appears to be a positive regulator, given that *Cab* is transcriptionally inactive in cells with nongreen plastids or with photooxidatively destroyed chloroplasts… It could easily be a small molecule produced by chloroplast metabolic activity.”

## Concluding remarks

This was a wise prediction since in the following 25 years several metabolic pathways in chloroplasts could be shown to be the origin of signals controlling the activities of nuclear genes. Impaired chloroplast transcription and/or chloroplast translation have striking impacts on the expression of nuclear genes (see above) and chloroplast proteins or RNAs are still today discussed as source of plastid signals (or more precisely as start points of signal transduction chains) to the nucleus. However, a block of chloroplast gene expression due to mutation, treatment with inhibitors or photodestruction exerts massive influences on chloroplast development, photosynthesis, pigment synthesis, and other metabolic pathways in the plastids. Such plants are very suitable to detect effects of plastid-to-nucleus signaling but less ideal for identifying specific signaling molecules and signaling pathways. Joan Chory and coworkers found a way to overcome this situation by treating transgenic Arabidopsis plants with norflurazon and screening for rare mutations that lead to the expression of reporter genes under the control of a *CAB/LHCP* promoter (Susek et al. 1993). In this and similar ways up to now six GUN (genomes uncoupled) mutants have been identified, five of which point specifically to the tetrapyrrole biosynthesis as source of molecules involved in plastid-to-nucleus signaling (Woodson et al. 2011). Plastid gene expression and tetrapyrrole biosynthesis can be regarded as source of “biogenic signals,” which report the developmental state of chloroplasts to the nucleus (Pogson et al. 2008). Further insights into plastid-to-nucleus signaling were provided by studies on specific metabolic processes and components. Singlet oxygen (Wagner et al. 2004), H_2_O_2_ (Maruta et al. 2012), the redox state of the photosynthetic electron transport chain (Pfalz et al. 2012), 3′-phosphoadenosine 5′-phosphate (Estavillo et al. 2011), the isoprenoid precursor methylerithritolcyclodiphosphate (Xiao et al. 2012), ß-cyclocitral (Ramel et al. 2012), and other potential candidates (Chi et al. 2015; Tian 2015; Chan et al. 2016; Kleine and Leister 2016; de Souza et al. 2017) were added to the list of “operational signals” (Pogson et al. 2008), which report changes in the status of the chloroplast in response to the environment to the nucleus. Changes in Ca^++^ levels might act as both biogenic and operational signal (Guo et al. 2016; de Souza et al. 2017). Also, hormones with plastids as major sites of their biosynthesis like cytokinins, ABA, jasmonic acid, and others are essential players in the interactions between plastids/chloroplasts and nucleus (Tian 2015; Chan et al. 2016) and coordinate the expression of nuclear and plastid genes during chloroplast development, senescence and in response to stress (Zubo et al. 2008, 2011; Yamburenko et al. 2015; Cortleven et al. 2016). Further components of the signal transduction pathways from plastids to the nucleus have been identified; in most cases, these processes remain poorly understood (Chan et al. 2016; Kleine and Leister 2016; de Souza et al. 2017).

Soon after its discovery, plastid-to-nucleus signaling was shown to affect not only the expression of nuclear genes encoding chloroplast-localized proteins as originally assumed (Bradbeer and Börner 1978; Hagemann and Börner 1978) but also the gene for nitrate reductase, a cytoplasmic enzyme (Börner et al. 1986; Oelmüller et al. 1988; Mohr et al. 1992; Hess et al. 1994). Somewhat later was reported that the expression of genes for peroxisomal enzymes (Schwartz et al. 1992; Boldt et al. 1997) and even mitochondrial DNA and RNA levels (Hedtke et al. 1999) are under control of plastid signal(s). The drastic increase in transcription and transcript levels of the gene(s) for chalcone synthase, a cytoplasmic enzyme regarded as fully unrelated to plastids/chloroplasts, as well as the enhanced expression of other stress- and defense-related genes in white albostrians leaves (Hess et al. 1994, 1998) were first indications for roles of plastid-to-nucleus signaling in the response of plants to biotic and abiotic stresses.

Research on plastid-to-nucleus signaling started with the observation of missing small subunits of Rubisco in plastid ribosome deficient mutants. Now, it is obvious that plastid signals are not only involved in harmonizing gene expression in plastids and nucleus for undisturbed chloroplast development. They are also generated to communicate with other regulatory networks and to alter the expression of thousands of genes at the level of transcription and posttranscriptional levels in order to adapt the metabolism inside the plastids and in other parts of the cell to environmental changes sensed by the plastids/chloroplasts (Chan et al. 2016; Kleine and Leister 2016; Woodson 2016; de Souza et al. 2017). Plastid-to-nucleus signaling that we once termed “chloroplast control principle” and speculated it might depend on a chloroplast gene product thereby giving the chloroplast some kind of autonomy (Hagemann and Börner 1978; Bradbeer and Börner 1978; Bradbeer et al. 1979) is, like replication and expression of chloroplast genes, completely under control of the nucleus.
